# Supplementary material for: Association of Clinical Aspects and Genetic Variants with the Severity of Cisplatin-Induced Ototoxicity in Head and Neck Squamous Cell Carcinoma: A Prospective Cohort Study
Source: Cancers (Basel). 2023 Mar 14;15(6):1759. doi: 10.3390/cancers15061759 (PMC10046479; doi:10.3390/cancers15061759)
Supplement: Supplementary file 1 [file cancers-15-01759-s001.zip › Table S1.pdf]

Table S1. Clinicopathological factors and minimum threshold averages for pure tone air conduction audiometry in high frequencies (3, 4, 6 and 8 kHz): linear regression coefficients.

Variable	Right ear		Left ear	
	Regression coefficient (95% CI)	p-value	Regression coefficient (9 CI)	p-value
**Univariate analysis**				
Age (years)	-0.38 (-0.81 to 0.04)	0.07	-0.11 (-0.55 to 0.32)	0.60
Sex (male)	-16.58 (-3.15 to -30)	0.02	-6.17 (-20.06 to 7.71)	0.38
Race (non-white)	-0.02 (-0.06 to 0)	0.12	-0.02 (-0.06 to 0)	0.20
Tobacco consumption	-1.56 (-25.6 to 23.50)	0.93	0.48 (-24.86 to 25.82)	0.97
Alcohol consumption	6.28 (-6.38 to 18.3)	0.32	-8.61 (-21.45 to 4.23)	0.18
ECOG	3.56 (-4.48 to 11.61)	0.38	6.36 (-1.59 to 14.32)	0.11
BMI	-0.87 (-1.75 to 0.01)	0.05	-1.02 (-1.89 to 0.14)	0.02
Diabetes	-2.16 (-14.55 to 10.23)	0.73	2.02 (-10.42 to 14.47)	0.74
Hypertension	0.09 (-8.32 to 8.52)	0.98	-4.49 (-12.90 to 3.91)	0.29
Tumor location	0.29 (-0.9 to 1.48)	0.62	-0.01 (-1.21 to 1.18)	0.98
Tumor side	1.34 (-4.47 to 7.16)	0.64	1.81 (-4.02 to 7.64)	0.53
Histological grade 3	0.29 (-10.85 to 10.85)	0.95	1.42 (-9.21 to 12.27)	0.79
Tumor stage	3.48 (-2.45 to 9.41)	0.24	5.30 (-0.59 to 11.20)	0.08
cCDDP dose (mg/m²)	0.08 (0.005 to 0.16)	0.03	0.04 (-0.04 to 0.12)	0.31
Radiotherapy dose (Gy)	0.17 (0.5 to 0.85)	0.61	0.34 (-0.34 to 1.32)	0.32
Supraclavicular fossa	0.002 (-0.002 to 0.007)	0.28	0.002 (-0.002 to 0.007)	0.35
Facial (right and left)	0.004 (-0.002 to 0.011)	0.24	0.002 (-0.004 to 0.01)	0.44
Boost (right and left)	0.006 (0.009 to 0.02)	0.41	0.003 (-0.01 to 0.02)	0.72
Posterior (right and left)	0.002 (-0.001 to 0.007)	0.23	0.001 (-0.003 to 0.006)	0.48
**Multivariate analysis**				
Age	-0.17 (-0.71 to 0.26)	0.41	NA	NA
BMI	-0.80 (-1.64 to 0.03)	0.06	-0.87 (-1.77 to 0.20)	0.06

Sex (male)	-17.07 (-3.95 to -30.2)	0.08	-3.83 (-9.83 to 2.16)	0.20
cCDDP dose (mg/m²)	0.08 (0.01 to 0.16)	0.02	NA	NA

BMI: body mass index; cCDDP: cumulative cisplatin; CI: confidence interval; ECOG: Eastern Cooperative Oncology Group performance status; Gy: Gray; mg: milligrams; NA: not applicable.
