# Supplementary material for: Association of Clinical Aspects and Genetic Variants with the Severity of Cisplatin-Induced Ototoxicity in Head and Neck Squamous Cell Carcinoma: A Prospective Cohort Study
Source: Cancers (Basel). 2023 Mar 14;15(6):1759. doi: 10.3390/cancers15061759 (PMC10046479; doi:10.3390/cancers15061759)
Supplement: Supplementary file 1 [file cancers-15-01759-s001.zip › Table S2.pdf]

Table S2. Single nucleotide variants encoding cisplatin metabolism and pure tone high frequency threshold averages in air conduction (3, 4, 6 and 8 KHz).

Variable	N	Right ear					Left ear				
		**Pretreatment Median (IQR)**	**Posttreatment Median (IQR)**	**Difference**	**p-value**	**PA (%)**	**Pretreatment Median (IQR)**	**Posttreatment Median (IQR)**	**Difference**	**p-value**	**PA (%)**
GSTM1											
Present	40	26.9 (17.5-48.4)	53.1 (29.1-68.8)	11.3 (3.1-30.9)	0.20	NA	23.8 (16.3-47.2)	53.1 (31.6-62.5)	15.0 (5.0-29.7)	0.10	NA
Null	49	37.5 (19.4-50.0)	61.3 (45.6-68.1)	20.0 (11.3-27.5)			35.0 (23.1-47.5)	60.0 (46.3-68.8)	22.5 (11.3-34.4)		
GSTT1											
Present	75	32.5 (17.5-50.0)	61.3 (43.8-68.8)	21.3 (7.5-31.3)	0.07	NA	30.0 (20.0-47.5)	57.5 (43.8-68.8)	20.0 (8.8-35.0)	0.04	59.4
Null	14	28.8 (20.-44.1)	31.3 (26.3-58.4)	10.0 (0.9-20.3)			35.0 (19.7-46.3)	43.1 (32.2-58.4)	11.3 (0.6-22.8)		
***GSTP1* c.313A>G**											
AA	40	31.3 (18.1-50.0)	55.6 (30.6-67.5)	16.3 (5.6-24.7)	0.43	NA	31.9 (19.1-47.2)	52.5 (39.1-64.1)	17.5 (7.8-25.6)	0.18	NA
AG or GG	49	27.5 (18.1-50.0)	61.3 (42.5-68.8)	20.0 (6.3-34.4)			28.8 (20.0-47.5)	58.8 (45.6-69.4)	20.0 (6.3-41.9)		
AA or AG	84	33.1 (17.8-50.0)	59.4 (38.8-68.8)	17.5 (5.6-26.3)	0.81	NA	33.1 (20.0-47.5)	55.0 (42.5-67.2)	18.8 (6.6-29.7)	0.37	NA
GG	5	21.3 (15.0-26.3)	45.0 (25.0-71.3)	30.0 (1.3-50.6)			16.3 (7.5-26.3)	40.0 (21.3-82.5)	30.0 (5.0-61.9)		
***XPC* c.2815A>C**											
AA	34	26.3 (16.9-42.2)	60.0 (43.4-67.8)	23.8 (10.0-39.4)	0.008	60.5	25.6 (18.8-46.3)	57.5 (40.0-68.8)	27.5 (8.8-40.3)	0.04	49.6
AC or CC	55	35.0 (20.0-51.3)	58.8 (32.5-68.8)	17.5 (5.0-22.5)			35.0 (20.0-48.8)	55.0 (41.3-65.0)	16.3 (6.3-26.3)		
AA or AC	77	28.8 (17.5-50.0)	58.8 (33.1-68.8)	17.5 (7.5-30.0)	0.16	NA	28.8 (19.4-47.5)	55.0 (41.3-66.3)	18.8 (8.8-33.8)	0.23	NA
CC	12	36.3 (22.8-52.2)	53.8 (41.3-68.4)	15.6 (0.0-22.5)			39.4 (25.0-48.4)	53.1 (36.9-70.3)	15.0 (4.1-25.6)		

***XPD* c.934G>A**											
GG	48	34.4 (19.1-51.3)	61.3 (44.1-69.7)	16.3 (5.0-30.9)	0.94	NA	33.8 (21.3-48.8)	57.5 (43.8-67.2)	18.1 (8.8-28.8)	0.81	NA
GA or AA	41	27.5 (17.5-46.3)	56.3 (32.5-67.5)	20.0 (10.6-26.3)			30.0 (18.8-45.6)	52.5 (36.9-67.5)	18.8 (5.0-33.8)		
GG or GA	81	32.5 (18.1-50.0)	58.8 (32.5-68.8)	17.5 (5.0-27.5)	0.12	NA	30.0 (20.0-48.1)	55.0 (40.6-66.3)	17.5 (6.9-29.4)	0.14	NA
AA	8	27.5 (18.-40.6)	53.1 (41.6-72.2)	21.9 (21.3-38.4)			31.9 (15.0-44.7)	58.1 (45.3-91.9)	35.0 (5.0-64.1)		
***XPD* c.2251A>C**											
AA	47	35.0 (18.8-51.3)	60.0 (45.0-68.8)	18.8 (7.5-28.8)	0.42	NA	33.8 (21.3-48.8)	55.0 (40.6-67.8)	16.3 (10.0-27.5)	1.00	NA
AC or CC	42	27.5 (17.5-48.1)	53.1 (30.9-67.5)	17.5 (3.4-30.0)			28.8 (18.1-47.5)	55.0 (41.3-66.3)	22.5 (3.4-36.9)		
AA or AC	80	29.4 (17.5-49.7)	60.0 (35.0-68.8)	18.1 (7.5-28.1)	0.38	NA	29.4 (20.0-47.2)	55.0 (40.3-67.2)	19.4 (9.1-30.0)	0.22	NA
CC	9	35.0 (23.1-52.5)	50.0 (35.6-75.0)	10.0 (11.3-35.6)			46.3 (20.6-55.6)	55.0 (47.5-73.8)	5.0 (1.9-50.0)		
***XPF* c.2505T>C**											
TT	40	28.1 (15.0-45.0)	58.8 (30.6-67.5)	21.3 (6.3-30.9)	0.47	NA	26.9 (20.0-46.3)	55.0 (33.1-65.9)	20. (7.2-35.9)	0.62	NA
TC or CC	49	32.5 (20.0-51.9)	58.8 (38.8-70.0)	17.5 (6.3-26.3)			35.0 (19.4-48.8)	57.5 (43.1-68.1)	16.3 (6.9-28.1)		
TT or TC	82	33.1 (18.4-50.3)	59.4 (39.7-68.8)	18.1 (5.0-29.1)	0.47	NA	31.3 (20.9-47.8)	56.3 (42.5-67.8)	18.1 (6.3-30.6)	0.39	NA
CC	7	20.0 (10.0-28.8)	38.8 (27.5-63.8)	17.5 (10.0-41.3)			16.3 (12.5-35.0)	41.3 (31.3-66.3)	22.5 (15.0-46.3)		
***ERCC1* c.354C>T**											
CC	22	28.8 (18.8-51.6)	57.5 (31.6-70.0)	14.4 (2.5-32.8)	0.82	NA	37.5 (20.6-48.8)	54.4 (42.5-68.4)	15.0 (5.0-33.1)	0.64	NA
CT or TT	67	32.5 (17.5-50.0)	58.8 (38.8-67.5)	18.8 (7.5-28.8)			28.8 (20.0-46.3)	55.0 (41.3-66.3)	20.0 (8.8-32.5)		
CC or CT	74	28.8 (18.8-50.3)	57.5 (32.2-67.8)	15.6 (5.0-26.9)	0.05	NA	29.4 (19.7-46.6)	54.4 (39.7-66.6)	18.8 (5.9-33.1)	0.80	NA
TT	15	35.0 (16.3-48.8)	65.0 (58.8-68.8)	22.5 (17.5-38.8)			40.0 (21.3-48.8)	60.0 (53.8-68.8)	20.0 (10.0-27.5)		
***MLH1* c.-93G>A**											

GG	52	35.0 (17.5-52.5)	63.8 (39.1-70.9)	18.1 (7.5-29.7)	0.51	NA	40.0 (20.3-50.9)	58.1 (42.5-68.8)	18.8 (6.3-31.9)	0.43	NA
GA or AA	37	27.5 (19.4-44.4)	52.5 (31.3-61.9)	17.5 (5.0-28.1)			27.5 (16.3-39.4)	53.8 (40.6-64.4)	18.8 (10.6-32.5)		
GG or GA	86	31.3 (18.4-50.0)	58.8 (33.4-68.8)	18.1 (5.0-29.1)	NE	NA	31.3 (20.0-47.5)	55.0 (40.9-67.8)	18.8 (6.3-30.6)	NE	NA
AA	3	26.3 (15.0-50.0)	61.3 (43.8-61.3)	17.5 (11.3-46.3)			25.0 (12.5-25.0)	53.8 (41.3-63.8)	28.8 (16.3-51.3)		
***MSH2* c.211+9G>C**											
GG	22	26.3 (12.8-44.7)	48.1 (38.1-73.9)	17.5 (4.1-35.9)	0.97	NA	26.9 (17.2-49.7)	51.9 (34.4-75.6)	20.6 (4.7-40.6)	0.54	NA
GC or CC	67	32.5 (20.0-50.0)	58.8 (33.8-68.8)	18.8 (7.5-26.3)			32.5 (20.0-47.5)	55.0 (45.0-66.3)	18.8 (8.8-28.8)		
GG or GC	69	28.8 (17.5-50.0)	60.0 (43.8-68.8)	20.0 (10.0-30.6)	0.22	NA	28.8 (19.4-47.5)	57.5 (42.5-68.1)	20.0 (8.1-36.9)	0.10	NA
CC	20	34.4 (20.3-50.6)	53.1 (30.0-68.4)	11.3 (5.0-23.4)			34.4 (20.0-48.4)	54.4 (33.1-65.9)	11.3 (5.3-24.7)		
***MSH3* c.3133A>G**											
AA	50	33.1 (20.0-50.3)	61.3 (33.4-69.1)	18.1 (5.0-27.2)	0.64	NA	34.4 (19.7-48.8)	57.5 (48.1-67.8)	18.8 (8.8-32.5)	0.67	NA
AG or GG	39	27.5 (13.8-48.8)	56.3 (40.0-65.0)	17.5 (7.5-31.3)			28.8 (20.0-46.3)	50.0 (48.1-67.8)	18.8 (5.0-30.0)		
AA or AG	82	32.5 (20.0-50.0)	60.0 (38.8-68.8)	17.5 (6.9-30.0)	0.93	NA	31.3 (20.0-47.5)	57.5 (42.5-67.8)	18.8 (7.2-33.1)	0.48	NA
GG	7	10.0 (3.8-48.8)	45.0 (25.0-46.3)	21.3 (1.3-28.8)			12.5 (7.5-48.8)	32.5 (27.5-53.8)	18.8 (5.0-23.8)		
***EXO1* c.1762G>A**											
GG	35	30.0 (17.5-50.0)	50.0 (30.0-67.5)	12.5 (5.0-23.8)	0.06	NA	30.0 (18.8-47.5)	53.8 (31.3-68.8)	15.0 (5.0-36.3)	0.32	NA
GA or AA	54	30.6 (18.8-50.0)	61.3 (44.7-69.1)	21.3 (10.0-32.5)			30.0 (20.9-47.8)	57.5 (43.4-66.6)	21.3 (9.7-30.6)		
GG or GA	79	30.0 (17.5-50.0)	57.5 (32.5-68.8)	17.5 (5.0-26.3)	0.08	NA	30.0 (18.8-47.5)	55.0 (40.0-66.3)	18.8 (6.3-28.8)	0.21	NA
AA	10	31.3 (19.7-51.3)	62.5 (56.6-70.6)	26.9 (16.9-41.3)			34.4 (23.1-51.3)	65.0 (52.8-77.2)	28.1 (9.7-41.6)		
***P53* c.215G>C**											
GG	40	35.0 (17.8-50.9)	60.6 (45.3-69.7)	18.1 (5.0-29.7)	0.46	NA	36.9 (19.1-50.6)	58.8 (40.6-69.7)	18.1 (5.3-27.2)	0.37	NA

GC or CC	49	27.5 (18.8-50.0)	57.5 (32.5-67.5)	17.5 (10.0-28.1)			28.8 (20.0-43.1)	53.8 (41.3-65.6)	18.8 (9.4-33.8)		
GG or GC	79	32.5 (17.5-50.0)	58.8 (33.8-68.8)	17.5 (5.0-26.3)	0.21	NA	30.0 (20.0-47.5)	55.0 (41.3-66.3)	17.5 (6.3-28.8)	0.06	NA
CC	10	23.8 (19.4-45.0)	60.0 (37.2-64.7)	23.8 (9.7-34.7)			27.5 (16.9-42.2)	55.6 (42.5-73.8)	32.5 (13.1-41.6)		
***FAS* c.-671A>G**											
AA	30	27.5 (16.9-45.0)	54.4 (30.9-64.1)	17.5 (8.8-30.6)	0.89	NA	25.0 (19.7-46.3)	52.5 (31.9-61.6)	18.8 (3.8-29.1)	0.23	NA
AG or GG	59	35.0 (18.8-51.3)	61.3 (38.8-70.0)	17.5 (5.0-28.8)			32.5 (20.0-48.8)	58.8 (42.5-68.8)	18.8 (10.0-35.0)		
AA or AG	66	28.1 (17.5-50.0)	58.1 (37.5-67.8)	18.8 (6.9-30.3)	0.69	NA	29.4 (19.7-47.5)	54.4 (40.0-66.3)	18.8 (6.3-29.1)	0.45	NA
GG	23	35.0 (20.0-50.0)	63.8 (32.5-68.8)	17.5 (2.5-26.3)			30.0 (21.3-47.5)	62.5 (42.5-68.8)	18.8 (7.5-52.5)		
***FAS* c.-1378G>A**											
GG	65	28.8 (18.1-51.3)	58.8 (41.9-68.8)	20.0 (5.0-30.6)	0.85	NA	30.0 (18.8-48.8)	57.5 (41.9-66.3)	18.8 (6.3-33.8)	0.90	NA
GA or AA	24	31.3 (17.8-46.9)	48.1 (30.3-68.4)	17.5 (10.3-25.6)			29.4 (21.6-45.0)	48.8 (40.3-69.7)	18.1 (8.8-27.5)		
GG or GA	84	32.5 (18.8-50.0)	58.8 (39.1-68.8)	19.4 (7.5-29.7)	0.91	NA	31.3 (20.0-47.5)	55.0 (42.5-67.2)	18.8 (7.8-31.9)	0.53	NA
AA	5	20.0 (5.0-38.8)	31.3 (15.6-63.1)	11.3 (0.6-34.4)			20.0 (10.0-38.8)	38.8 (13.8-67.5)	8.8 (0.6-38.1)		
***FASL* c.-844C>T**											
CC	28	26.9 (20.0-40.3)	56.9 (40.0-68.8)	21.3 (12.8-30.9)	0.27	NA	29.4 (16.9-40.0)	55.0 (41.3-64.7)	19.4 (11.3-27.2)	0.61	NA
CT or TT	61	35.0 (17.5-51.3)	61.3 (33.1-68.8)	17.5 (5.0-27.5)			30.0 (20.6-48.8)	55.0 (41.3-68.8)	18.8 (5.0-33.8)		
CC or CT	66	33.1 (18.8-51.3)	60.0 (45.0-70.0)	19.4 (6.9-31.6)	0.38	NA	34.4 (20.0-49.1)	57.5 (45.9-67.8)	19.4 (8.4-30.6)	0.66	NA
TT	23	26.3 (17.5-42.5)	38.8 (25.0-67.5)	12.5 (5.0-25.0)			28.8 (18.8-40.0)	46.3 (27.5-66.3)	16.3 (5.0-35.0)		
***CASP3* c.-1191A>G**											
AA	36	30.6 (20.0-50.0)	59.4 (35.0-66.9)	18.8 (8.1-30.0)	0.60	NA	28.8 (20.3-48.8)	56.3 (42.8-68.1)	23.1 (7.8-33.8)	0.17	NA
AG or GG	53	30.0 (16.9-50.0)	58.8 (35.6-68.8)	17.5 (5.0-27.5)			30.0 (17.5-46.9)	55.0 (40.0-66.9)	16.3 (5.0-31.3)		

AA or AG	79	28.8 (17.5-50.0)	58.8 (33.8-67.5)	17.5 (7.5-28.8)	1.00	NA	28.8 (18.8-46.3)	55.0 (40.0-66.3)	18.8 (6.3-32.5)	0.86	NA
GG	10	40.6 (24.7-60.9)	68.8 (51.3-73.4)	20.0 (0.3-35.9)			43.8 (21.9-70.6)	63.8 (50.9-85.0)	15.0 (7.8-32.2)		
***CASP3* c.-182-247G>T**											
GG	33	27.5 (20.0-47.5)	50.0 (30.6-68.1)	11.3 (1.9-27.5)	0.05	NA	28.8 (20.6-47.5)	52.5 (36.9-65.6)	15.0 (4.4-30.6)	0.29	NA
GT or TT	56	33.1 (17.5-50.0)	61.3 (44.1-68.8)	20.0 (11.3-30.0)			33.8 (18.8-47.5)	57.5 (44.1-68.8)	20.0 (7.8-31.9)		
GG or GT	78	31.3 (18.8-50.3)	58.8 (33.4-68.8)	17.5 (5.0-29.1)	0.30	NA	30.0 (20.0-47.8)	55.0 (41.3-68.8)	16.9 (6.3-32.5)	0.78	NA
TT	11	28.8 (11.3-43.8)	60.0 (40.0-63.8)	21.3 (15.0-32.5)			33.8 (11.3-40.0)	57.5 (30.0-63.8)	20.0 (7.5-26.3)		

N: number of patients; PA: power analysis; IQR: interquartile range; NA: not applied. Multiple linear regression with audiometric patterns were adjusted for cumulative cisplatin dose.
