# Supplementary material for: Association of Clinical Aspects and Genetic Variants with the Severity of Cisplatin-Induced Ototoxicity in Head and Neck Squamous Cell Carcinoma: A Prospective Cohort Study
Source: Cancers (Basel). 2023 Mar 14;15(6):1759. doi: 10.3390/cancers15061759 (PMC10046479; doi:10.3390/cancers15061759)
Supplement: Supplementary file 1 [file cancers-15-01759-s001.zip › Table S3.pdf]

Table S3. Combinations of single nucleotide variants encoding cisplatin metabolism and their association with pure tone high-frequency threshold averages (3, 4, 6, and 8 KHz)

Variable	N	Right ear					Left ear				
		Pretreatment Median (IQR)	Posttreatment Median (IQR)	Difference	*P*-value	PA (%)	Pretreatment Median (IQR)	Posttreatment Median (IQR)	Difference	*P*-value	PA (%)
***GSTM1 + XPC* c.2815A>C**											
Present + CC	6	27.5 (17.8-63.1)	44.4 (23.8-67.5)	3.1 (5.0-24.1)	0.06	NA	32.5 (15.0-55.6)	43.8 (25.0-60.6)	20.9 (5.0-27.5)	0.02	59
Null + AA or AC	43	36.3 (18.8-50.0)	61.3 (43.8-67.5)	20.0 (12.5-30.0)			33.8 (21.3-33.8)	58.8 (46.3-68.8)	36.3 (20.0-49.3)		
***GSTM1 + XPC* c.2815A>C**											
Present + AC or CC	22	26.9 (16.3-51.6)	42.5 (25.9-61.3)	6.3 (0.6-22.2)	0.01	59	24.4 (15.0-48.8)	51.3 (29.4-58.8)	6.3 (3.4-16.9)	0.03	51
Null + AA	16	23.1 (11.9-44.1)	58.8 (47.2-64.1)	22.5 (6.3-37.2)			28.8 (20.3-46.3)	63.1 (41.3-68.8)	27.5 (5.0-40.0)		
***GSTM1 + EXO1* c.1762G>A**											
Present + GG	13	21.3 (18.8-48.1)	31.3 (25.6-63.8)	5.0 (2.5-18.8)	0.008	75	23.8 (17.5-48.1)	38.8 (28.8-60.0)	8.8 (5.0-15.0)	0.005	85
Null + GA or AA	27	37.5 (21.3-50.0)	62.5 (57.5-68.8)	21.3 (16.3-30.0)			40.0 (25.0-48.8)	61.3 (52.5-68.8)	22.5 (11.3-28.8)		
***GSTM1 + P53* c.215G>C**											
Present + GG or GC	35	27.5 (17.5-50.0)	57.5 (30.0-72.5)	11.3 (2.5-31.3)	0.06	NA	23.8 (16.3-46.3)	53.8 (32.5-62.5)	15.0 (5.0-28.8)	0.02	57
Null + CC	5	26.3 (20.6-48.1)	62.5 (60.0-66.3)	32.5 (18.1-41.3)			28.8 (18.8-36.3)	66.3 (51.9-88.1)	40.0 (26.9-56.9)		
***GSTM1 + FASL* c.-844C>T**											
Present + TT	13	26.3 (17.5-42.5)	31.3 (26.9-56.3)	11.3 (3.1-18.1)	0.05	54	23.8 (17.5-46.9)	38.8 (28.8-58.1)	15.0 (4.4-18.1)	0.07	NA
Null + CC or CT	39	37.5 (20.0-51.3)	61.3 (46.3-68.8)	18.8 (10.0-26.3)			40.0 (23.8-52.5)	58.8 (48.8-70.0)	20.0 (11.3-27.5)		
***GSTT1 + XPD* c.2251A>C**											
Present + AA	41	35.0 (17.5-51.3)	61.3 (46.3-70.6)	21.3 (7.5-30.6)	0.05	61	33.8 (22.5-48.8)	55.0 (43.1-67.5)	17.5 (9.4-28.1)	0.04	56

Null + AC or CC	8	28.8 (21.9-41.6)	31.3 (27.2-54.4)	6.9 (0.9-20.9)			35.0 (20.3-44.7)	43.1 (35.9-54.4)	5.0 (3.1-23.4)		
***GSTT1* + *MSH2* c.211+9G>C**											
Present + GG or GC	60	28.8 (16.6-50.0)	61.3 (45.0-68.8)	21.3 (10.0-32.5)	0.09	NA	28.8 (19.1-47.5)	57.5 (44.4-68.8)	21.3 (9.1-39.7)	0.04	62
Null + CC	5	30.0 (20.6-43.8)	30.0 (25.6-43.1)	5.0 (11.3-13.1)			30.0 (18.1-50.0)	38.8 (31.3-50.0)	8.8 (0.0-13.1)		
***GSTP1* c.313A>G + *XPC* c.2815A>C**											
AA or AG + AC or CC	54	35.6 (20.0-51.6)	58.8 (32.5-68.8)	17.5 (5.0-23.1)	0.15	NA	36.9 (20.9-48.8)	55.0 (42.2-65.6)	16.3 (6.3-26.3)	0.03	60
GG + AA	4	23.1 (12.5-26.3)	47.5 (36.6-81.9)	32.5 (13.1-58.4)			18.1 (5.0-27.5)	53.1 (34.4-90.6)	41.9 (19.7-65.9)		
***GSTP1* c.313A>G + *XPC* c.2815A>C**											
AA + AC or CC	25	28.8 (18.8-46.9)	50.0 (30.0-65.6)	17.5 (7.5-23.8)	0.005	88	33.8 (17.5-42.5)	52.5 (38.1-62.5)	16.3 (8.8-23.8)	0.01	76
AG or GG + AA	19	20.0 (15.0-35.0)	61.3 (50.0-67.5)	30.0 (16.3-48.8)			23.8 (13.8-41.3)	62.5 (42.5-68.8)	38.8 (16.3-52.5)		
***GSTP1* c.313A>G + *XPD* c.934G>A**											
AA + GG or GA	37	32.5 (18.8-50.0)	58.8 (29.4-67.5)	15.0 (5.0-25.6)	0.02	65	30.0 (18.8-47.5)	52.5 (39.4-63.1)	16.3 (8.1-26.9)	0.01	62
AG or GG + AA	5	26.3 (14.4-38.8)	56.3 (50.0-78.1)	30.0 (21.3-53.8)			28.8 (8.1-43.1)	66.3 (47.5-103.1)	53.8 (22.5-68.8)		
***GSTP1* c.313A>G + *XPD* c.2251A>C**											
AA + AA	22	35.6 (24.7-51.3)	58.8 (46.3-71.6)	19.4 (6.9-26.3)	0.92	NA	34.4 (24.7-49.1)	55.0 (43.1-66.3)	16.3 (9.7-26.6)	0.45	NA
AG or GG + AC or CC	24	27.5 (19.1-51.9)	59.4 (45.9-68.4)	20.6 (3.1-42.2)			28.1 (20.0-51.3)	63.1 (52.5-69.7)	27.5 (3.1-44.4)		
***GSTP1* c.313A>G + *MSH3* c.3133A>(**											
AA + AG or GG	19	27.5 (12.5-48.8)	46.3 (26.3-67.5)	15.0 (7.5-25.0)	0.71	NA	26.3 (12.5-40.0)	47.5 (31.3-65.0)	18.8 (3.8-27.5)	0.24	NA
AG or GG + AA	29	27.5 (19.4-50.0)	65.0 (33.1-70.0)	20.0 (4.4-38.1)			28.8 (19.4-51.9)	63.8 (53.1-72.5)	20.0 (7.5-46.9)		
***GSTP1* c.313A>G + *EXO1* c.1762G>A**											
AA + GG or GA	37	30.0 (17.5-49.4)	50.0 (29.4-67.5)	15.0 (5.0-24.4)	0.06	NA	30.0 (18.8-45.6)	48.8 (36.9-63.1)	18.8 (8.1-25.0)	0.03	59.8
AG or GG + AA	7	26.3 (18.8-43.8)	63.8 (50.0-70.0)	33.8 (20.0-41.3)			28.8 (21.3-55.0)	68.8 (50.0-87.5)	32.5 (16.3-46.3)		
***GSTP1* c.313A>G + *P53* c.215G>C**											

AA + GG	14	46.9 (15.6-58.4)	59.4 (41.6-71.6)	8.8 (3.4-23.4)	0.23	NA	41.9 (20.9-59.7)	56.3 (43.4-67.8)	15.0 (5.9-23.8)	0.08	NA
AG or GG + GC or CC	23	27.5 (17.5-52.5)	61.3 (40.0-68.8)	20.0 (5.0-41.3)			28.8 (20.0-48.8)	57.5 (52.5-68.8)	27.5 (11.3-42.5)		
***GSTP1* c.313A>G + *FAS* c.-671A>G**											
AA + AA or AG	30	28.8 (17.5-55.9)	55.6 (36.3-64.7)	16.3 (5.0-26.3)	0.67	NA	31.9 (19.7-47.8)	53.1 (38.8-66.3)	20.0 (9.7-27.5)	0.04	56
AG or GG + GG	13	37.5 (19.4-51.3)	67.5 (47.5-71.9)	21.3 (0.6-39.4)			30.0 (21.3-48.1)	66.3 (58.8-88.1)	38.8 (13.1-53.8)		
***GSTP1* c.313A>G + *FASL* c.-844C>T**											
AA + CT or TT	25	32.5 (17.5-50.6)	58.8 (26.9-67.5)	11.3 (5.0-23.1)	0.13	NA	30.0 (19.4-48.8)	52.5 (31.9-66.3)	16.3 (5.6-28.1)	0.18	NA
AG or GG + CC	13	25.0 (18.8-39.4)	60.0 (38.8-70.0)	22.5 (13.8-41.3)			21.3 (15.0-40.6)	58.8 (42.5-68.8)	23.8 (11.3-46.9)		
***XPC* c.2815A>C + *XPD* c.2251A>C**											
AC or CC + AA	27	36.3 (17.5-50.0)	60.0 (43.8-68.8)	18.8 (7.5-26.3)	0.07	NA	35.0 (21.3-48.8)	55.0 (41.3-68.8)	15.0 (10.0-26.3)	0.07	NA
AA + AC or CC	14	19.4 (10.9-31.3)	56.9 (36.6-67.5)	30.0 (10.9-49.4)			22.5 (12.2-31.3)	63.8 (37.2-70.0)	36.9 (6.6-52.8)		
***XPC* c.2815A>C + *ERCC1* c.354C>T**											
CC + CC or CT	12	36.3 (22.8-52.2)	53.8 (41.3-68.4)	15.6 (0.0-22.5)	0.05	41	39.4 (25.0-48.4)	53.1 (36.9-70.3)	15.0 (4.1-25.6)	0.35	NA
AA or AC + TT	15	35.0 (16.3-48.8)	65.0 (58.8-68.8)	22.5 (17.5-38.8)			40.0 (21.3-48.8)	60.0 (53.8-68.8)	20.0 (10.0-27.5)		
***XPC* c.2815A>C + *ERCC1* c.354C>T**											
AC or CC + CC or CT	46	33.8 (20.0-52.5)	51.3 (30.9-68.8)	14.4 (4.7-22.5)	0.002	61	34.4 (19.7-49.7)	53.8 (39.7-65.6)	15.0 (5.0-26.6)	0.33	NA
AA + TT	6	26.3 (10.3-52.5)	65.0 (55.6-72.5)	34.4 (16.6-54.1)			36.3 (17.2-50.3)	58.8 (53.1-69.4)	18.8 (8.1-46.9)		
***XPC* c.2815A>C + *MSH3* c.3133A>G**											
AC or CC + AG or GG	23	28.8 (12.5-48.8)	46.3 (27.5-65.0)	15.0 (5.0-22.5)	0.10	NA	32.5 (12.5-47.5)	48.8 (31.3-65.0)	13.8 (5.0-22.5)	0.10	NA
AA + AA	18	27.5 (17.5-41.3)	60.0 (32.8-68.8)	22.5 (9.4-34.7)			29.4 (17.2-46.9)	58.8 (39.7-69.4)	18.8 (8.8-52.5)		
***XPC* c.2815A>C + *EXO1* c.1762G>A**											
AC or CC + GG	26	38.8 (20.0-52.5)	45.6 (30.0-67.8)	11.9 (2.5-21.6)	0.004	64	34.4 (19.7-49.7)	53.1 (31.3-64.1)	15.0 (5.0-22.8)	0.05	49
AA + GA or AA	25	28.8 (18.8-50.6)	61.3 (45.6-70.0)	23.8 (10.0-36.9)			26.3 (19.4-47.5)	57.5 (41.3-66.3)	27.5 (10.6-39.4)		

***XPC* c.2815A>C + *EXO1* c.1762G>A**										
CC + GG or GA	11	35.0 (21.3-52.5)	48.8 (40.0-70.0)	21.3 (1.3-22.5)	0.04	51	38.8 (25.0-47.5)	52.5 (35.0-71.3)	18.8 (3.8-26.3)	0.12	NA
AA or AC + AA	9	26.3 (19.4-47.5)	63.8 (54.4)	32.5 (20.0-41.3)			28.8 (22.5-47.5)	66.3 (55.0-80.6)	32.5 (13.1-43.1)		
***XPC* c.2815A>C + *EXO1* c.1762G>A**										
AC or CC + GG or GA	50	33.1 (20.0-50.6)	55.0 (30.9-68.8)	16.3 (4.7-22.5)	0.03	50	33.8 (20.0-47.5)	54.4 (39.7-65.0)	15.6 (5.9-24.4)	0.07	NA
AA + AA	5	26.3 (19.4-38.8)	61.3 (46.3-70.0)	32.5 (14.4-45.0)			26.3 (22.5-39.4)	66.3 (50.0-71.3)	40.0 (13.1-46.3)		
***XPC* c.2815A>C + *P53* c.215G>C**										
AC or CC + GG	21	43.8 (20.6-51.9)	60.0 (38.8-70.0)	17.5 (4.4-22.5)	0.03	51	45.0 (22.5-54.4)	62.5 (40.0-69.4)	15.0 (5.0-23.1)	0.04	63
AA + GC or CC	15	28.8 (17.5-41.3)	58.8 (38.8-67.5)	23.8 (11.3-38.8)			28.8 (20.0-41.3)	60.0 (40.0-66.3)	28.8 (10.0-40.0)		
***XPC* c.2815A>C + *P53* c.215G>C**										
CC + GG or GC	10	36.3 (25.9-55.9)	53.8 (40.3-71.3)	8.8 (2.2-22.5)	0.06	NA	39.4 (25.0-50.6)	53.1 (40.6-68.4)	8.1 (1.6-24.4)	0.03	69
AA or AC + CC	8	23.8 (20.3-49.7)	60.0 (32.8-66.3)	20.6 (6.6-39.1)			27.5 (19.1-44.7)	55.6 (46.9-68.1)	32.5 (11.9-40.0)		
***XPC* c.2815A>C + *P53* c.215G>C**										
AC or CC + GG or GC	49	35.0 (20.0-50.6)	58.8 (31.9-68.8)	17.5 (5.0-22.5)	0.03	41	38.8 (21.9-50.6)	55.0 (41.9-66.3)	16.3 (5.6-25.0)	0.01	67
AA + CC	4	23.1 (18.1-37.5)	60.0 (36.3-65.9)	29.4 (15.0-39.1)			27.5 (20.6-47.5)	67.5 (53.1-83.8)	37.5 (31.3-40.0)		
***XPC* c.2815A>C + *FAS* c.-1378G>A**										
AC or CC + GG	39	32.5 (20.0-52.5)	58.8 (32.5-68.8)	15.0 (3.8-25.0)	0.04	18	33.8 (18.8-52.5)	55.0 (41.3-65.0)	15.0 (6.3-27.5)	0.03	38
AA + GA or AA	8	23.1 (17.8-31.9)	41.9 (29.1-66.9)	17.5 (8.1-45.0)			23.8 (19.4-29.7)	48.1 (39.1-79.1)	26.9 (12.5-49.7)		
***XPC* c.2815A>C + *FAS* c.-671A>G**										
AC or CC + AA	15	27.5 (20.0-43.8)	48.8 (30.0-62.5)	15.0 (5.0-25.0)	0.13	NA	25.0 (16.3-40.0)	52.5 (30.0-57.5)	18.8 (3.8-23.8)	0.03	55
AA + AG or GG	19	26.3 (18.8-41.3)	61.3 (46.3-72.5)	30.0 (7.5-41.3)			26.3 (13.8-41.3)	65.0 (40.0-71.3)	35.0 (11.3-52.5)		
***XPC* c.2815A>C + *FAS* c.-671A>G**										
AC or CC + AA or AG	41	27.5 (17.5-50.6)	52.5 (30.6-66.9)	17.5 (5.0-25.6)	0.06	NA	32.5 (18.1-48.1)	53.8 (33.1-65.0)	16.3 (5.6-25.0)	0.04	42

AA + GG	9	20.0 (14.4-33.8)	61.3 (29.4-70.0)	30.0 (6.3-50.0)			21.3 (11.9-30.0)	65.0 (35.6-71.3)	38.8 (9.4-53.8)		
***XPC* c.2815A>C + *FASL* c.-844C>T**											
AC or CC + TT	12	28.8 (13.8-44.7)	38.1 (25.0-66.6)	13.8 (6.6-22.2)	0.02	56	26.3 (12.5-40.0)	38.8 (27.5-64.4)	15.0 (5.3-26.6)	0.12	NA
AA + CC or CT	23	26.3 (15.0-50.0)	61.3 (56.3-72.5)	30.0 (11.3-48.8)			23.8 (13.8-46.3)	57.5 (45.0-68.8)	27.5 (10.0-45.0)		
***XPC* c.2815A>C + *FASL* c.-844C>T**											
AC or CC + CT or TT	35	43.8 (17.5 -52.5)	58.8 (31.3-68.8)	15.0 (2.5-22.5)	0.003	76	40.0 (20.0-52.5)	55.0 (46.3-68.8)	15.0 (5.0-27.5)	0.02	60
AA + CC	8	22.5 (14.7-37.5)	56.9 (47.2-72.5)	30.6 (17.5-50.9)			16.3 (12.5-38.1)	58.8 (43.8-70.0)	34.4 (16.6-53.4)		
***XPC* c.2815A>C+*CASP3* c.-182-247G**											
CC + CC	7	27.5 (21.3-52.5)	46.3 (26.3-70.0)	10.0 (1.3-22.5)	0.04	31	38.8 (18.8-47.5)	48.8 (35.0-67.5)	11.3 (5.0-23.8)	0.08	NA
AA or AC + CA or AA	51	32.5 (17.5-50.0)	61.3 (43.8-68.8)	20.0 (11.3-30.0)			30.0 (18.8-47.5)	57.5 (43.8-68.8)	20.0 (8.8-32.5)		
***XPC* c.2815A>C+*CASP3* c.-182-247G**											
AC or CC + CC	22	31.3 (20.0-50.6)	49.4 (30.0-68.8)	13.1 (0.6-25.3)	0.007	64	30.6 (20.9-48.8)	52.5 (39.7-65.6)	15.0 (3.8-27.8)	0.07	NA
AA + CA or AA	23	26.3 (13.8-45.0)	61.3 (50.0-68.8)	26.3 (11.3-48.8)			25.0 (18.8-46.3)	57.5 (45.0-68.8)	28.8 (8.8-45.0)		
***XPD* c.934G>A + *EXO1* c.1762G>A**											
GG + GG or GA	42	34.4 (17.5-51.6)	61.3 (40.3-69.1)	14.4 (4.7-29.1)	0.24	NA	33.8 (19.7-48.8)	56.3 (42.5-66.6)	19.4 (8.1-28.8)	0.04	57
GA or AA + AA	4	31.3 (11.3-53.1)	65.6 (53.4-74.1)	30.6 (20.0-46.9)			30.6 (8.1-51.3)	68.8 (53.4-84.1)	39.4 (25.9-50.9)		
***XPD* c.934G>A + *CASP3* c.-182-247G**											
GG or GA + CC	30	27.5 (20.0-46.3)	52.5 (29.7-67.8)	10.6 (1.3-26.9)	0.04	41	28.1 (17.5-43.1)	53.1 (37.8-65.3)	15.0 (4.7-29.7)	0.08	NA
AA + CA or AA	5	26.3 (18.8-38.8)	56.3 (44.4-78.1)	21.3 (21.3-48.1)			28.8 (17.5-43.1)	66.3 (45.6-103.1)	53.8 (11.3-68.8)		
***XPD* c.934G>A + *FAS* c.-1378G>A**											
GG or GA + GG	61	32.5 (18.1-51.3)	60.0 (41.9-68.8)	17.5 (5.0-30.0)	0.35	NA	30.0 (19.4-49.4)	57.5 (41.9-66.9)	18.8 (6.3-32.5)	0.05	52
AA + GA or AA	4	31.3 (18.1-50.0)	56.9 (41.6-72.2)	21.9 (21.3-28.1)			34.4 (24.1-45.6)	85.0 (52.5-105.3)	45.6 (23.8-69.4)		
***XPD* c.934G>A + *FAS* c.-671A>G**											

GG or GA + AA	27	27.5 (15.0-45.0)	52.5 (30.0-63.8)	15.0 (5.0-32.5)	0.06	NA	23.8 (18.8-40.0)	52.5 (30.0-61.3)	18.8 (5.0-30.0)	0.05	90
AA + AG or GG	5	20.0 (13.1-34.4)	50.0 (44.4-78.1)	30.0 (21.3-53.8)			22.5 (8.1-34.4)	66.3 (48.1-103.1)	53.8 (35.0-68.8)		
***XPD* c.934G>A + *FASL* c.-844C>T**											
GG or GA + TT	21	26.3 (15.6-42.5)	33.8 (25.0-67.5)	12.5 (5.0-25.6)	0.03	57	26.3 (17.5-36.3)	46.3 (27.5-66.3)	16.3 (5.6-32.5)	0.11	NA
AA + CC or CT	6	23.1 (15.3-39.4)	53.1 (47.2-79.4)	26.3 (21.3-47.5)			25.6 (10.3-46.6)	58.1 (45.9-78.1)	35.0 (17.5-57.8)		
***XPD* c.2251A>C + *EXO1* c.1762G>A**											
AA + GG or GA	41	35.0 (17.5-50.6)	60.0 (44.4-68.8)	17.5 (6.3-26.3)	0.28	NA	33.8 (20.0-46.9)	55.0 (40.6-65.6)	16.3 (10.6-27.5)	0.03	62
AC or CC + AA	4	31.3 (11.3-53.1)	65.6 (53.4-74.1)	30.6 (20.0-46.9)			30.6 (8.1-51.3)	68.8 (53.4-84.1)	39.4 (25.9-50.9)		
***XPD* c.2251A>C + *CASP3* c.-1191A>[illegible]**											
AA or AC + AA	32	33.1 (20.0-50.0)	60.0 (35.3-66.9)	18.8 (8.1-29.1)	0.29	NA	28.1 (20.3-48.8)	56.3 (42.8-68.1)	23.1 (9.1-29.7)	0.05	55
CC + AG or GG	5	50.0 (21.9-75.6)	56.3 (41.3-75.6)	5.0 (11.3-31.3)			52.5 (25.0-66.9)	52.5 (47.5-69.4)	5.0 (3.1-25.6)		
***ERCC1* c.354C>T + *EXO1* c.1762G>[illegible]**											
CC or CT + GG	32	31.3 (18.1-51.9)	45.6 (30.0-63.1)	11.9 (3.1-22.2)	0.05	51	31.3 (19.1-47.2)	52.5 (31.3-65.0)	15.0 (4.1-23.8)	0.78	NA
TT + GA or AA	12	39.4 (18.8-49.7)	64.4 (49.4-67.8)	20.6 (17.5-29.1)			42.5 (22.2-49.7)	58.8 (44.4-64.7)	18.1 (9.1-25.6)		
***ERCC1* c.354C>T + *FASL* c.-844C>T**											
CC or CT + TT	21	26.3 (17.5-41.9)	38.8 (26.9-65.6)	12.5 (6.3-25.0)	0.02	59	28.8 (19.4-37.5)	46.3 (28.8-67.5)	16.3 (5.6-35.6)	0.53	NA
TT + CC or CT	13	35.0 (16.9-49.4)	65.0 (59.4-70.0)	22.5 (18.1-43.8)			40.0 (22.5-49.4)	60.0 (55.6-69.4)	22.5 (10.0-30.0)		
***ERCC1* c.354C>T + *FASL* c.-844C>T**											
CC or CT + CT or TT	53	30.0 (17.5-51.9)	58.8 (31.9-68.1)	15.0 (5.0-27.5)	0.03	60	28.8 (20.0-48.8)	53.8 (39.4-68.8)	18.8 (5.0-35.6)	0.29	NA
TT + CC	7	26.3 (16.3-43.8)	63.8 (46.3-72.5)	26.3 (20.0-48.8)			25.0 (18.8-40.0)	60.0 (41.3-63.8)	26.3 (16.3-32.5)		
***MLH1* c.-93G>A + *CASP3* c.-182-247[illegible]**											
GG + CC	18	31.9 (19.4-52.5)	57.5 (37.5-70.6)	10.6 (3.1-29.1)	0.05	NA	36.9 (23.1-52.5)	55.6 (38.8-69.4)	13.8 (3.8-28.8)	0.05	20
GA or AA + CA or AA	22	28.1 (17.8-45.9)	56.9 (44.7-61.6)	21.3 (10.3-32.2)			29.4 (17.2-42.5)	54.4 (43.1-65.9)	20.0 (10.9-32.8)		

***MSH2* c.211+9G>C + *EXO1* c.1762G>**												
GG + GG	8	47.5 (23.8-80.9)	54.4 (35.6-74.1)	7.5 (6.9-16.3)	0.02	72	44.4 (24.7-67.5)	67.5 (41.6-100.6)	10.0 (4.1-45.0)	0.60	NA
GC or CC + GA or AA	40	36.9 (26.3-51.3)	63.1 (53.4-70.9)	20.0 (8.1-26.3)			39.4 (22.8-48.8)	57.5 (49.7-66.3)	20.0 (9.1-28.4)		
***EXO1* c.1762G>A + *P53* c.215G>C**											
GG + GG or GC	31	28.8 (17.5-45.0)	50.0 (30.0-67.5)	12.5 (5.0-25.0)	0.02	60	28.8 (18.8-47.5)	55.0 (31.3-68.8)	15.0 (3.8-36.3)	0.03	50
GA or AA + CC	6	20.6 (15.0-30.0)	60.0 (37.2-63.8)	32.5 (22.5-41.3)			22.5 (11.3-35.0)	61.9 (44.1-73.8)	37.5 (27.2-41.6)		
***EXO1* c.1762G>A + *CASP3*c.-182-247**											
GG + GG	14	39.4 (20.0-52.5)	45.6 (28.8-68.8)	7.5 (1.3-22.5)	0.002	79	31.3 (19.1-48.8)	47.5 (29.1-63.1)	11.3 (1.9-23.8)	0.05	NA
GA or AA + GT or TT	35	37.5 (18.8-51.3)	62.5 (52.5-70.0)	21.3 (11.3-32.5)			38.8 (18.8-48.8)	57.5 (46.3-66.3)	20.0 (8.8-30.0)		
***EXO1* c.1762G>A + *FASL* c.-844C>T**											
GG + TT	12	25.0 (14.7-44.7)	30.6 (24.1-66.6)	11.3 (3.1-19.7)	0.04	57	26.3 (17.2-38.1)	35.0 (24.7-60.3)	11.3 (4.1-15.9)	0.15	NA
GA or AA + CC or CT	43	33.8 (18.8-51.3)	61.3 (48.8-70.0)	20.0 (10.0-33.8)			33.8 (21.3-48.8)	57.5 (46.3-65.0)	20.0 (8.8-28.8)		
***EXO1* c.1762G>A + *FASL* c.-844C>T**											
GG or GA + TT	20	29.4 (14.7-43.4)	35.0 (25.0-67.5)	11.9 (5.0-23.4)	0.04	46	29.4 (16.9-40.0)	42.5 (27.5-65.9)	15.0 (4.1-29.7)	0.35	NA
AA + CC or CT	7	43.8 (18.8-51.3)	67.5 (58.8-72.5)	21.3 (20.0-41.3)			48.8 (21.3-55.0)	63.8 (53.8-87.5)	23.8 (8.8-23.8)		
***EXO1* c.1762G>A + *FASL* c.-844C>T**											
GG + CT or TT	25	35.0 (16.3-52.5)	56.3 (30.0-68.1)	12.5 (1.3-23.1)	0.04	62	32.5 (20.0-50.61)	53.8 (35.0-66.9)	15.0 (4.4-36.9)	0.35	NA
GA or AA + CC	18	27.5 (20.3-41.9)	60.0 (45.6-70.0)	21.9 (15.6-35.0)			31.9 (13.4-42.8)	57.5 (43.1-64.1)	20.6 (11.3-28.8)		
***P53* c.215G>C + *CASP3* c.-182-247G>**											
GG + CC	14	25.6 (18.4-55.9)	51.3 (25.6-70.0)	6.3 (1.3-25.9)	0.02	50	24.4 (15.6-53.4)	51.9 (19.7-74.7)	11.3 (3.4-24.7)	0.12	NA
GC or CC + CA or AA	30	26.9 (17.5-50.6)	58.1 (31.9-67.8)	17.5 (11.3-27.8)			26.9 (17.2-46.6)	54.4 (40.9-66.3)	18.8 (10.3-33.1)		
***CASP3* c.-1191A>G + *FASL* c.-844C>**											
AA or AG + TT	19	26.3 (13-41.3)	33.8 (25.0-63.8)	11.3 (5.0-23.8)	0.73	NA	26.3 (18.8-35.0)	40.0 (27.5-66.3)	16.3 (3.8-36.3)	0.94	NA

GG + CC or CT	6	43.1 (23.4-86.6)	69.4 (51.3-86.3)	17.5 (8.1-42.5)			63.1 (21.9-82.5)	73.1 (60.0-99.7)	18.1 (2.5-42.5)		
***CASP3* c.-182-247G>T + *FAS* c.-671A** [illegible]											
CC + AA	9	27.5 (17.5-40.6)	38.8 (21.3-57.5)	10.0 (0.0-21.9)	0.06	NA	25.0 (18.1-43.8)	35.0 (16.9-60.0)	3.8 (3.8-25.6)	0.03	53
CA or AA + AG or GG	35	37.5 (17.5-52.5)	61.3 (45.0-71.3)	18.8 (7.5-26.3)			40.0 (18.8-48.8)	63.8 (46.3-73.8)	20.0 (7.5-35.0)		
***CASP3* c.-182-247G>T+*FASL* c.-844** [illegible]											
CC + CT or TT	23	27.5 (18.8-45.0)	48.8 (30.0-67.5)	10.0 (1.3-26.3)	0.03	57	27.5 (20.0-40.0)	52.5 (32.5-65.0)	16.3 (5.0-36.3)	0.19	NA
CA or AA + CC	18	23.8 (17.5-38.4)	55.0 (37.2-62.8)	21.9 (13.8-32.2)			25.6 (12.5-39.1)	56.3 (40.9-64.1)	23.1 (15.9-32.2)		

PA: power analysis; IQR: interquartile range; NA: not applied. Multiple linear regression with audiometric patterns were adjusted for cumulative cisplatin dose
