# Supplementary material for: Association of Clinical Aspects and Genetic Variants with the Severity of Cisplatin-Induced Ototoxicity in Head and Neck Squamous Cell Carcinoma: A Prospective Cohort Study
Source: Cancers (Basel). 2023 Mar 14;15(6):1759. doi: 10.3390/cancers15061759 (PMC10046479; doi:10.3390/cancers15061759)
Supplement: Supplementary file 1 [file cancers-15-01759-s001.zip › Table S4.pdf]

Table S4. Clinical characteristics of patients stratified by intensity of ototoxicity classified by NCI CTCAE v4.0 criteria

Variable	Ototoxicity			
	Grade 0-2 Median (IQR), N(%)	Grade 3 Median (IQR), N(%)	OR (95%CI)	p-value
**Univariate analysis**				
Age (years)	58 (51-62)	54 (47-57)	0.97 (0.92-1.0)	0.26
Gender (male)	59 (93.7)	23 (88.5)	0.52 (0.10-2.5)	0.41
Race (non-white)	3 (4.8)	6 (23)	6.0 (1.37-26.2)	0.01
Tobacco consumption	61 (96.8)	26 (100.0)	NA	0.36*
Alcohol consumption	56 (88.9)	25 (96.2)	3.12 (0.36-32.76)	0.29
ECOG			1.57 (0.61-4.04)	0.34
0	43 (68.3)	15 (57.6)		
1	20 (31.7)	11 (42.4)		
BMI	19.7 (17.2-23.3)	17.8 (15.6-20.3)	0.86 (0.76-0.98)	0.03
Diabetes	6 (9.5%)	3 (11.5%)	1.23 (0.28-5.37)	0.77
Hypertension	18 (28.6%)	6 (23.1%)	0.75 (0.25-2.17)	0.59
Tumor location	40 (63.5%)	15 (57.7%)	1.06 (0.91-1.23)	0.39
Tumor side			1.17 (0.58-2.37)	0.64
Right	26 (41.9)	11 (42.3)		
Left	31 (50)	11 (42.3)		
Medial	5 (8.1%)	4 (15.4)		
Differentiation grade 3	10 (18.2)	3 (17.6)	0.96 (0.23-4.0)	0.96
Tumor stage (AJCC)			4.0 (0.9-16.2)	0.18
I	1 (1.6)	0 (0.0)		
II	5 (7.9)	0 (0.0)		
III	12 (19.0)	2 (7.7)		
IV	45 (71.4)	24 (92.3)		
cCDDP dose (mg/m^2)	260 (200-300)	260 (240-280)	1.0 (0.99-1.01)	0.66
**Multivariate analysis**				
Race (non-white)			5.43 (1.21-24.27)	0.02
BMI			0.89 (0.72-0.98)	0.03

AJCC: American Joint Committee in Cancer; BMI: body mass index; cCDDP: cumulative cisplatin; CI: confidence interval; ECOG: Eastern Cooperative Oncology Group performance status; NA: not applicable; OR: odds ratio. *Since the proportion of smokers was close to 100% in this study, it was not possible to estimate odds ratio; in this case.
