# Supplementary material for: Association of Clinical Aspects and Genetic Variants with the Severity of Cisplatin-Induced Ototoxicity in Head and Neck Squamous Cell Carcinoma: A Prospective Cohort Study
Source: Cancers (Basel). 2023 Mar 14;15(6):1759. doi: 10.3390/cancers15061759 (PMC10046479; doi:10.3390/cancers15061759)
Supplement: Supplementary file 1 [file cancers-15-01759-s001.zip › Table S5.pdf]

Table S5. Single nucleotide variants and hearing impairment (according to NCI criteria - NCI CTCAE v4.0) related to cisplatin-based chemoradiation.

Variable	N	Ototoxicity				
		Grade 0-2 N(%)	Grade 3 N(%)	OR (95% CI)	p-value	PA (%)
GSTM1						
Present	40	29 (46.0)	11 (42.3)	Reference		
Null	49	34 (54.0)	15 (57.7)	1.30 (0.47-3.59)	0.60	NA
GSTT1						
Present	75	51 (81.0)	24 (92.3)	Reference		
Null	14	12 (19.0)	2 (7.7)	0.47 (0.09-24.05)	0.36	NA
***GSTP1* c.313A>G**						
AA	40	33 (52.4)	7(26.9)	Reference	**0.01**[1]	65
AG or GG	49	30 (47.6)	19 (73.1)	4.20 (1.34-13.16)		
AA or AG	84	60 (95.2)	24 (92.3)	Reference	0.57	NA
GG	5	3 (4.8)	2 (7.7)	1.72 (0.25-11.64)		
***XPC* c.2815A>C**						
AA	34	18 (28.6)	16 (61.5)	3.13 (1.27-7.70)	**0.01**[2]	65
AC or CC	55	45 (71.4)	10 (38.5)	Reference		
AA or AC	77	53 (84.1)	24 (92.3)	Reference	0.57	NA
CC	12	10 (15.9)	2 (7.7)	0.62 (0.12-3.26)		
***XPD* c.934G>A**						
GG	48	33 (52.4)	15 (57.7)	Reference	0.51	NA
GA or AA	41	30 (47.6)	11 (42.3)	0.71 (0.26-1.93)		
GG or GA	81	59 (93.7)	22 (84.6)	Reference	0.21	NA
AA	8	4 (6.3)	4 (15.4)	2.66 (0.56-12.57)		
***XPD* c.2251A>C**						
AA	47	34 (54.0)	13 (50.0)	Reference	0.68	NA
AC or CC	42	29 (46.0)	13 (50.0)	1.22 (0.46-3.25)		
AA or AC	80	57 (90.5)	23 (88.5)	Reference	0.42	NA
CC	9	6 (9.5)	3 (11.5)	1.89 (0.39-9.10)		

***XPF* c.2505T>C**						
TT	40	28 (44.4)	12 (46.2)	Reference	0.75	NA
TC or CC	49	35 (55.6)	14 (53.8)	0.85 (0.31-2.29)		
TT or TC	82	58 (92.1)	24 (92.3)	Reference	0.81	NA
CC	7	5 (7.9)	2 (7.7)	1.24 (0.20-7.41)		
***ERCC1* c.354C>T**						
CC	22	15 (23.8)	7 (26.9)	Reference	0.90	NA
CT or TT	67	48 (76.2)	19 (73.1)	1.07 (0.34-3.35)		
CC or CT	74	52 (82.5)	22 (84.6)	Reference	0.84	NA
TT	15	11 (17.5)	4 (15.4)	1.14 (0.31-4.22)		
***MLH1* c.-93G>A**						
GG	52	35 (55.6)	17 (65.4)	Reference	0.43	NA
GA or AA	37	28 (44.4)	9 (34.6)	0.65 (0.23-1.87)		
GG or GA	86	61 (96.8)	25 (96.2)	Reference	0.55	NA
AA	3	2 (3.2)	1 (3.8)	2.15 (0.16-27.58)		
***MSH2* c.211+9G>C**						
GG	22	14 (22.2)	8 (30.8)	Reference	0.36	NA
GC or CC	67	49 (77.8)	18 (69.2)	0.60 (0.20-1.80)		
GG or GC	69	47 (74.6)	22 (84.6)	Reference	0.35	NA
CC	20	16 (25.4)	4 (15.4)	0.54 (0.15-1.96)		
***MSH3* c.3133A>G**						
AA	50	35 (55.6)	15 (57.7)	Reference	0.35	NA
AG or GG	39	28 (44.4)	11 (42.3)	0.61 (0.22-1.71)		
AA or AG	82	56 (88.9)	26 (100.0)	NE	NE	NA
GG	7	7 (11.1)	0 (0.0)			
***EXO1* c.1762G>A**						
GG	35	27 (42.9)	8 (30.8)	Reference	0.47	NA
GA or AA	54	36 (57.1)	18 (69.2)	1.46 (0.52-4.12)		
GG or GA	79	59 (93.7)	20 (76.9)	Reference	0.13	NA
AA	10	4 (6.3)	6 (23.1)	3.00 (0.71-12.73)		
***P53* c.215G>C**						
GG	40	29 (46.0)	11 (42.3)	Reference	0.84	NE

GC or CC	49	34 (54.0)	15 (57.7)	1.10 (0.41-2.94)		
GG or GC	79	59 (93.7)	20 (76.9)	Reference	0.09	NA
CC	10	4 (6.3)	6 (23.1)	3.63 (0.81-16.21)		
***FAS* c.-671A>G**						
AA	30	22 (34.9)	8 (30.8)	Reference	0.91	NA
AG or GG	59	41 (65.1)	18 (69.2)	1.06 (0.36-3.06)		
AA or AG	66	46 (73.0)	20 (76.9)	Reference	0.41	NA
GG	23	17 (27.0)	6 (23.1)	0.61 (0.19-1.98)		
***FAS* c.-1378G>A**						
GG	65	46 (73.0)	19 (73.1)	Reference	0.50	NA
GA or AA	24	17 (27.0)	7 (26.9)	0.67 (0.21-2.10)		
GG or GA	84	59 (93.7)	25 (96.2)	Reference	0.72	NA
AA	5	4 (6.3)	1 (3.8)	0.65 (0.06-6.57)		
***FASL* c.-844C>T**						
CC	28	20 (31.7)	8 (30.8)	Reference	0.78	NA
CT or TT	61	43 (68.3)	18 (69.2)	0.86 (0.30-2.46)		
CC or CT	66	45 (71.4)	21 (80.8)	Reference	0.11	NA
TT	23	18 (28.6)	5 (19.2)	0.33 (0.08-1.32)		
***CASP3* c.-1191A>G**						
AA	36	23 (36.5)	13 (50.0)	Reference	0.52	NA
AG or GG	53	40 (63.5)	13 (50.0)	0.72 (0.26-1.95)		
AA or AG	79	57 (90.5)	22 (84.6)	Reference	0.39	NA
GG	10	6 (9.5)	4 (15.4)	1.91 (0.42-8.53)		
***CASP3* c.-182-247G>T**						
GG	33	24 (38.1)	9 (34.6)	Reference	0.82	NA
GT or TT	56	39 (61.9)	17 (65.4)	1.12 (0.40-3.16)		
GG or GT	78	54 (85.7)	24 (92.3)	Reference	0.62	NA
TT	11	9 (14.3)	2 (7.7)	0.65 (0.12-3.50)		

N: number of patients; NCI: National Cancer Institute; PA: power analysis; G: grade; OR: odds ratio; CI: confidence interval; IQR: interquartile range; NA: not applied. Logistic multivariate regression in hearing impairment (ototoxicity) was adjusted by race and body mass index. [1]p bootstrap=0.01; [2]p bootstrap=0.007
