# Supplementary material for: Association of Clinical Aspects and Genetic Variants with the Severity of Cisplatin-Induced Ototoxicity in Head and Neck Squamous Cell Carcinoma: A Prospective Cohort Study
Source: Cancers (Basel). 2023 Mar 14;15(6):1759. doi: 10.3390/cancers15061759 (PMC10046479; doi:10.3390/cancers15061759)
Supplement: Supplementary file 1 [file cancers-15-01759-s001.zip › Table S6.pdf]

Table S6. Associations for single nucleotide variants and hearing impairment (according to NCI criteria - NCI CTCAE v4.0) related to cisplatin-based chemoradiation.

Variable	N	Ototoxicity Grade 0-2 N(%)	Grade 3 N(%)	OR (95% CI)	p-value	PA (%)
***GSTM1 + XPC* c.2815A>C**						
Present + CC	6	5 (14.7)	1 (6.7)	Reference	0.66	NA
Null + AA or AC	43	29 (85.3)	14 (93.3)	1.68 (0.16-17.30)		
***GSTM1 + XPC* c.2815A>C**						
Present + AC or CC	22	19 (70.4)	3 (27.3)	Reference	0.02[1]	99
Null + AA	16	8 (29.6)	8 (72.7)	8.19 (1.28-52.20)		
***GSTM1 + EXO1* c.1762G>A**						
Present + GG	13	12 (38.7)	1 (11.1)	Reference	0.08	NA
Null + GA or AA	27	19 (61.3)	8 (88.9)	9.70 (0.74-126.01)		
***GSTM1 + P53* c.215G>C**						
Present + GG or GC	35	26 (96.3)	9 (69.2)	Reference	0.05	NA
Null + CC	5	1 (3.7)	4 (30.8)	11.53 (0.92-144.35)		
***GSTM1 + FASL* c.-844C>T**						
Present + TT	13	11 (28.9)	2 (14.3)	Reference	0.16	NA
Null + CC or CT	39	27 (71.1)	12 (85.7)	4.81 (0.52-44.07)		
***GSTT1 + XPD* c.2251A>C**						
Present + AA	41	29 (80.6)	12 (92.3)	Reference	0.48	NA
Null + AC or CC	8	7 (19.4)	1 (7.7)	0.45 (0.04-4.21)		
***GSTT1 + MSH2* c.211+9G>C**						
Present + GG or GC	60	40 (88.9)	20 (100.0)	NE	NE	NA
Null + CC	5	5 (11.1)	0 (0.0)			
***GSTP1* c.313A>G + *XPC* c.2815A>C**						
AA or AG + AC or CC	54	44 (95.7)	10 (83.3)	Reference	0.22	NA
GG + AA	4	2 (4.3)	2 (16.7)	3.70 (0.44-31.07)		
***GSTP1* c.313A>G + *XPC* c.2815A>C**						
AA + AC or CC	25	24 (72.7)	1 (9.1)	Reference	0.004[2]	97
AG or GG + AA	19	9 (27.3)	10 (90.9)	32.22 (3.09-335.52)		
***GSTP1* c.313A>G + *XPD* c.934G>A**						
AA + GG or GA	37	30 (96.8)	7 (63.6)	Reference	0.02[3]	92

AG or GG + AA	5	1 (3.2)	4 (36.4)	19.44 (1.59-237.72)		
***GSTP1* c.313A>G + *XPD* c.2251A>C**						
AA + AA	22	18 (56.3)	4(28.6)	Reference	0.03[4]	54
AG or GG + AC or CC	24	14 (43.8)	10 (71.4)	6.35 (1.09-36.92)		
***GSTP1* c.313A>G + *MSH3* c.3133A>G**						
AA + AG or GG	19	15 (46.9)	4 (25.0)	Reference	0.04[5]	54
AG or GG + AA	29	17 (53.1)	12 (75.0)	5.99 (1.08-33.09)		
***GSTP1* c.313A>G + *EXO1* c.1762G>A**						
AA + GG or GA	37	31 (93.9)	6 (54.5)	Reference	0.01[6]	81
AG or GG + AA	7	2 (6.1)	5 (45.5)	12.08 (1.60-91.01)		
***GSTP1* c.313A>G + *P53* c.215G>C**						
AA + GG	14	12 (48.0)	2 (16.7)	Reference	0.04[7]	64
AG or GG + GC or CC	23	13 (52.0)	10 (83.3)	8.08 (1.06-61.29)		
***GSTP1* c.313A>G + *FAS* c.-671A>G**						
AA + AA or AG	30	23 (76.7)	7 (53.8)	Reference	0.23	NE
AG or GG + GG	13	7 (23.3)	6 (46.2)	2.52 (0.55-11.52)		
***GSTP1* c.313A>G + *FASL* c.-844C>T**						
AA + CT or TT	25	20 (74.1)	5 (45.5)	Reference	0.03[8]	52
AG or GG + CC	13	7 (25.9)	6 (54.5)	7.91 (1.17-53.13)		
***XPC* c.2815A>C + *XPD* c.2251A>C**						
AC or CC + AA	27	22 (78.6)	5 (38.5)	Reference	0.01[9]	63
AA + AC or CC	14	6 (21.4)	8 (61.5)	8.79 (1.45-53.24)		
***XPC* c.2815A>C + *ERCC1* c.354C>T**						
CC + CC or CT	12	10 (47.6)	2 (33.3)	NA	NA	NA
AA or AC + TT	15	11 (52.4)	4 (66.7)			
***XPC* c.2815A>C + *ERCC1* c.354C>T**						
AC or CC + CC or CT	46	37 (92.5)	9 (75.0)	Reference	0.08	NA
AA + TT	6	3 (7.5)	3 (25.0)	5.26 (0.79-34.89)		
***XPC* c.2815A>C + *MSH3* c.3133A>G**						
AC or CC + AG or GG	23	21 (65.6)	2 (22.2)	Reference	0.009[10]	88
AA + AA	18	11 (34.4)	7 (77.8)	17.09 (2.02-144.32)		
***XPC* c.2815A>C + *EXO1* c.1762G>A**						
AC or CC + GG	26	23 (62.2)	3 (21.4)	Reference	0.02[11]	60
AA + GA or AA	25	14 (37.8)	11 (78.6)	6.32 (1.23-32.38)		

***XPC* c.2815A>C + *EXO1* c.1762G>A**						
CC + GG or GA	11	9 (75.0)	2 (25.0)	Reference	0.39	NA
AA or AC + AA	9	3 (25.0)	6 (75.0)	3.02 (0.24-37.85)		
***XPC* c.2815A>C + *EXO1* c.1762G>A**						
AC or CC + GG or GA	50	43 (95.6)	7 (70.0)	Reference	0.07	NA
AA + AA	5	2 (4.4)	3 (30.0)	6.65 (0.81-54.51)		
***XPC* c.2815A>C + *P53* c.215G>C**						
AC or CC + GG	21	19 (70.4)	2 (22.2)	Reference	0.03[12]	78
AA + GC or CC	15	8 (29.6)	7 (77.8)	17.66 (1.24-249.83)		
***XPC* c.2815A>C + *P53* c.215G>C**						
CC + GG or GC	10	9 (75.0)	1 (16.7)	Reference	0.07	NA
AA or AC + CC	8	3 (25.0)	5 (83.3)	14.46 (0.74-279.26)		
***XPC* c.2815A>C + *P53* c.215G>C**						
AC or CC + GG or GC	49	41 (100.0)	8 (66.7)	NA	NA	NA
AA + CC	4	0 (0.0)	4 (33.3)			
***XPC* c.2815A>C + *FAS* c.-1378G>A**						
AC or CC + GG	39	31 (91.2)	8 (61.5)	Reference	0.07	NA
AA + GA or AA	8	3 (8.8)	5 (38.5)	4.69 (0.83-26.35)		
***XPC* c.2815A>C + *FAS* c.-671A>G**						
AC or CC + AA	15	12 (60.0)	3 (21.4)	Reference	0.08	NA
AA + AG or GG	19	8 (40.0)	11 (78.6)	4.37 (0.81-23.46)		
***XPC* c.2815A>C + *FAS* c.-671A>G**						
AC or CC + AA or AG	41	33 (86.8)	8 (66.7)	Reference	0.20	NA
AA + GG	9	5 (13.2)	4 (33.3)	2.92 (0.55-15.56)		
***XPC* c.2815A>C + *FASL* c.-844C>T**						
AC or CC + TT	12	11 (50.0)	1 (7.7)	Reference	0.01[13]	82
AA + CC or CT	23	11 (50.0)	12 (92.3)	22.29 (1.79-276.99)		
***XPC* c.2815A>C + *FASL* c.-844C>T**						
AC or CC + CT or TT	35	28 (90.3)	7 (58.3)	Reference	0.03[14]	55
AA + CC	8	3 (9.7)	5 (41.7)	7.05 (1.17-42.47)		
***XPC* c.2815A>C + *CASP3* c.-182-247G>T**						
CC + CC	7	6 (14.6)	1 (5.9)	Reference	0.45	NA
AA or AC + CA or AA	51	35 (85.4)	16 (94.1)	2.31 (0.25-21.26)		
***XPC* c.2815A>C+ *CASP3* c.-182-247G>T**						

AC or CC + CC	22	17 (60.7)	5 (29.4)	Reference	0.06	NA
AA + CA or AA	23	11 (39.3)	12 (70.6)	3.87 (0.93-16.03)		
***XPD* c.934G>A + *EXO1* c.1762G>A**						
GG + GG or GA	42	30 (96.8)	12 (80.0)	Reference	0.13	NA
GA or AA + AA	4	1 (3.2)	3 (20.0)	6.50 (0.57-73.32)		
***XPD* c.934G>A + *CASP3* c.-182-247G>T**						
GG or GA + CC	30	22 (91.7)	8 (72.7)	Reference	0.43	NA
AA + CA or AA	5	2 (8.3)	3 (27.3)	2.57 (0.23-27.77)		
***XPD* c.934G>A + *FAS* c.-1378G>A**						
GG or GA + GG	61	44 (95.7)	17 (89.5)	Reference	0.41	NA
AA + GA or AA	4	2 (4.3)	2 (10.5)	2.42 (0.28-20.79)		
***XPD* c.934G>A + *FAS* c.-671A>G**						
GG or GA + AA	27	19 (95.0)	8 (66.7)	Reference	0.12	NA
AA + AG or GG	5	1 (5.0)	4 (33.3)	8.01 (0.56-113.81)		
***XPD* c.934G>A + *FASL* c.-844C>T**						
GG or GA + TT	21	17 (85.0)	4 (57.1)	Reference	0.39	NA
AA + CC or CT	6	3 (15.0)	3 (42.9)	2.98 (0.24-36.20)		
***XPD* c.2251A>C + *EXO1* c.1762G>A**						
AA + GG or GA	41	31 (96.9)	10 (76.9)	Reference	0.11	NA
AC or CC + AA	4	1 (3.1)	3 (23.1)	6.91 (0.61-77.93)		
***XPD* c.2251A>C + *CASP3* c.-1191A>G**						
AA or AC + AA	32	21 (84.0)	11 (91.7)	Reference	0.74	NA
CC + AG or GG	5	4 (16.0)	1 (8.3)	1.56 (0.10-23.47)		
***ERCC1* c.354C>T + *EXO1* c.1762G>A**						
CC or CT + GG	32	26 (72.2)	6 (75.0)	Reference	0.59	NA
TT + GA or AA	12	10 (27.8)	2 (25.0)	0.60 (0.09-3.84)		
***ERCC1* c.354C>T + *FASL* c.-844C>T**						
CC or CT + TT	21	16 (64.0)	5 (55.6)	Reference	0.45	NA
TT + CC or CT	13	9 (36.0)	4 (44.4)	1.92 (0.34-10.75)		
***ERCC1* c.354C>T + *FASL* c.-844C>T**						
CC or CT + CT or TT	53	36 (90.0)	17 (85.0)	Reference	0.42	NA
TT + CC	7	4 (10.0)	3 (15.0)	2.01 (0.35-11.24)		
***MLH1* c.-93G>A + *CASP3* c.-182-247G>T**						
GG + CC	18	12 (42.9)	6 (50.0)	Reference	0.67	NA

GA or AA + CA or AA	22	16 (57.1)	6 (50.0)	0.73 (0.17-3.08)		
***MSH2* c.211+9G>C + *EXO1* c.1762G>A**						
GG + GG	8	6 (17.6)	2 (14.3)	Reference	0.78	NA
GC or CC + GA or AA	40	28 (82.4)	12 (85.7)	1.32 (0.18-9.58)		
***EXO1* c.1762G>A + *P53* c.215G>C**						
GG + GG or GC	31	24 (96.0)	7 (58.3)	Reference	0.01[15]	85
GA or AA + CC	6	1 (4.0)	5 (41.7)	20.97 (1.66-264.08)		
***EXO1* c.1762G>A + *CASP3*c.-182-247G>T**						
GG + GG	14	13 (34.2)	1 (9.1)	Reference	0.16	NA
GA or AA + GT or TT	35	25 (65.8)	10 (90.9)	5.95 (0.48-73.27)		
***EXO1* c.1762G>A + *FASL* c.-844C>T**						
GG + TT	12	11 (27.5)	1 (6.7)	Reference	0.06	NA
GA or AA + CC or CT	43	29 (72.5)	14 (93.3)	12.64 (0.84-189.32)		
***EXO1* c.1762G>A + *FASL* c.-844C>T**						
GG or GA + TT	20	17 (85.0)	3 (42.9)	Reference	0.06	NA
AA + CC or CT	7	3 (15.0)	4 (57.1)	11.45(0.88-148.86)		
***EXO1* c.1762G>A + *FASL* c.-844C>T**						
GG + CT or TT	25	19 (61.3)	6 (50.0)	Reference	0.62	NA
GA or AA + CC	18	12 (38.7)	6 (50.0)	1.41 (0.35-5.63)		
***P53* c.215G>C + *CASP3* c.-182-247G>T**						
GG + CC	14	10 (33.3)	4 (28.6)	Reference	0.99	NA
GC or CC + CA or AA	30	20 (66.7)	10 (71.4)	1.01 (0.23-4.37)		
***CASP3* c.-1191A>G + *FASL* c.-844C>T**						
AA or AG + TT	19	14 (87.5)	5 (55.6)	Reference	0.03	65.9
GG + CC or CT	6	2 (12.5)	4 (44.4)	32.2 (1.18-874.27)		
***CASP3* c.-182-247G>T + *FAS* c.-671A>G**						
CC + AA	9	7 (22.6)	2 (15.4)	Reference	0.88	NA
CA or AA + AG or GG	35	24 (77.4)	11 (84.6)	1.14 (0.17-7.39)		
***CASP3* c.-182-247G>T+*FASL* c.-844C>T**						
CC + CT or TT	23	17 (56.7)	6 (54.5)	Reference	0.66	NA
CA or AA + CC	18	13 (43.3)	5 (45.5)	1.40 (0.30-6.54)		

N: number of patients, PA: power analysis, G: grade of ototoxicity, OR: odds ratio (adjusted), CI: confidence interval, NA: not applied. Multivariate analysis in hearing impairment (ototoxicity) was adjusted by race and body mass index. [1]p bootstrap=0.01; [2]p

bootstrap=0.009; [3]p bootstrap=0.002; [4]p bootstrap=0.03; [5]p bootstrap=0.02; [6]p bootstrap=0.005; [7]p bootstrap=0.01; [8]p bootstrap=0.01; [9]p bootstrap=0.003; [10]p bootstrap=0.001; [11]p bootstrap=0.02; [12]p bootstrap=0.01; [13]p bootstrap=0.005; [14]p bootstrap=0.01; [15]p bootstrap=0.005
